# Clinical and Functional Outcomes Following Repeat VA-ECMO in Adults: A Propensity Score–Matched Comparative Study

**DOI:** 10.3390/jcm15176627

**Published:** 2026-08-27

**Authors:** Kostiantyn Kozakov, Alois Philipp, Maik Foltan, Henrik Heuer, Walter Petermichl, Alexander Dietl, Bernhard Flörchinger, Leopold Rupprecht, Simon Schopka, Christof Schmid, Zdenek Provaznik

**Affiliations:** 1 Department of Cardiothoracic Surgery, University Medical Center Regensburg, Franz-Josef-Strauß-Allee 11, 93053 Regensburg, Germanyzdenek.provaznik@klinik.uni-regensburg.de (Z.P.); 2 Department of Anesthesiology, University Medical Center Regensburg, Franz-Josef-Strauß-Allee 11, 93053 Regensburg, Germany; 3 Department of Internal Medicine II, University Medical Center Regensburg, Franz-Josef-Strauß-Allee 11, 93053 Regensburg, Germany

**Keywords:** ECLS, second run, repeat VA-ECMO, survival, functional outcome, ECOG, MOF

## Abstract

**Background/Objectives**: Evidence on outcomes after repeat VA-ECMO in adults remains limited. This study aimed to compare survival and post-discharge functional outcomes between adults requiring a second VA-ECMO run and propensity-matched patients supported with a single VA-ECMO run and to characterize the clinical course during repeat support. **Methods**: This is an observational, single-center, retrospective cohort study with a propensity score–matched comparative analysis, conducted at a university tertiary-care referral center between 2006 and 2024. This study included 1496 adult patients (≥18 years) treated with VA-ECMO, of whom 30 (2.0%) required a second VA-ECMO run. Primary endpoints were in-hospital mortality, 30-day outcomes, and overall survival. Secondary exploratory analyses assessed baseline hemodynamic and metabolic parameters, multiorgan failure, neurological complications, and cannulation-related complications. **Results**: Repeat VA-ECMO was associated with longer support duration (median 6 vs. 4 days; *p* = 0.003) and a higher risk of MOF (OR 3.45; 95% CI 1.45–8.19; *p* = 0.003). No statistically significant differences in survival were observed (in-hospital mortality (OR 1.01; 95% CI, 0.48–2.11; *p* = 0.984); 30-day survival (OR 1.49; 95% CI, 0.72–3.07; *p* = 0.280); overall survival (OR 1.27; 95% CI, 0.59–2.73; *p* = 0.272). Repeat VA-ECMO was associated with higher transfusion requirements (RBC median 7.5 vs. 2 units; *p* < 0.001). After propensity matching, prolonged support, increased MOF, and higher transfusion burden persisted. ECOG outcomes were comparable overall; however, a borderline trend toward worse functional status was observed in patients undergoing repeat VA-ECMO during the same hospitalization (*p* = 0.057). **Conclusions**: Repeat VA-ECMO may be feasible in carefully selected adults, with no significant differences in survival, but is associated with prolonged support, increased transfusion requirements, and substantially higher MOF risk.

## 1. Introduction

Veno-arterial extracorporeal membrane oxygenation (VA-ECMO) serves as a temporary therapy to support patients with refractory cardiac and/or respiratory failure. Its growing utilization reflects improvements in technology, increased biocompatibility, and the expansion of specialized ECLS (extracorporeal life support) centers [1]. However, ECMO therapy entails substantial risks and high resource demands, rendering a highly selective, closely monitored and time-limited therapeutic approach appropriate [2,3]. The complexity is further amplified when VA-ECMO is re-initiated in the same patient after a previous successful weaning, posing additional clinical, ethical, and prognostic challenges. The feasibility of repeated successful weaning, the potential accumulation of ECMO-related complications, and the risk of further deterioration of the patient’s baseline condition raise critical uncertainty as to whether outcomes following repeated ECMO runs can approximate those achieved after a single course of veno-arterial ECMO. Taken together, these factors make clinical decision-making much more difficult.

The use of repeated courses of VA-ECMO in neonatal and pediatric populations has been well documented [4,5,6,7]. In adult patients, however, available studies report heterogeneous and partly conflicting outcomes [8,9,10,11,12]. Furthermore, despite increasing clinical experience with repeated ECMO support, the existing evidence for adults is largely limited to survival analyses in small cohorts [9], providing restricted insight into post-discharge functional status and lacking detailed characterization of the clinical course during a second VA-ECMO run.

In the present study we sought to evaluate both survival outcomes and post-discharge functional status using the ECOG performance scale and to systematically compare clinical characteristics and support between patients undergoing a second VA-ECMO run and those treated with a single ECMO course.

## 2. Materials and Methods

### 2.1. Study Design

**Figure 1:**
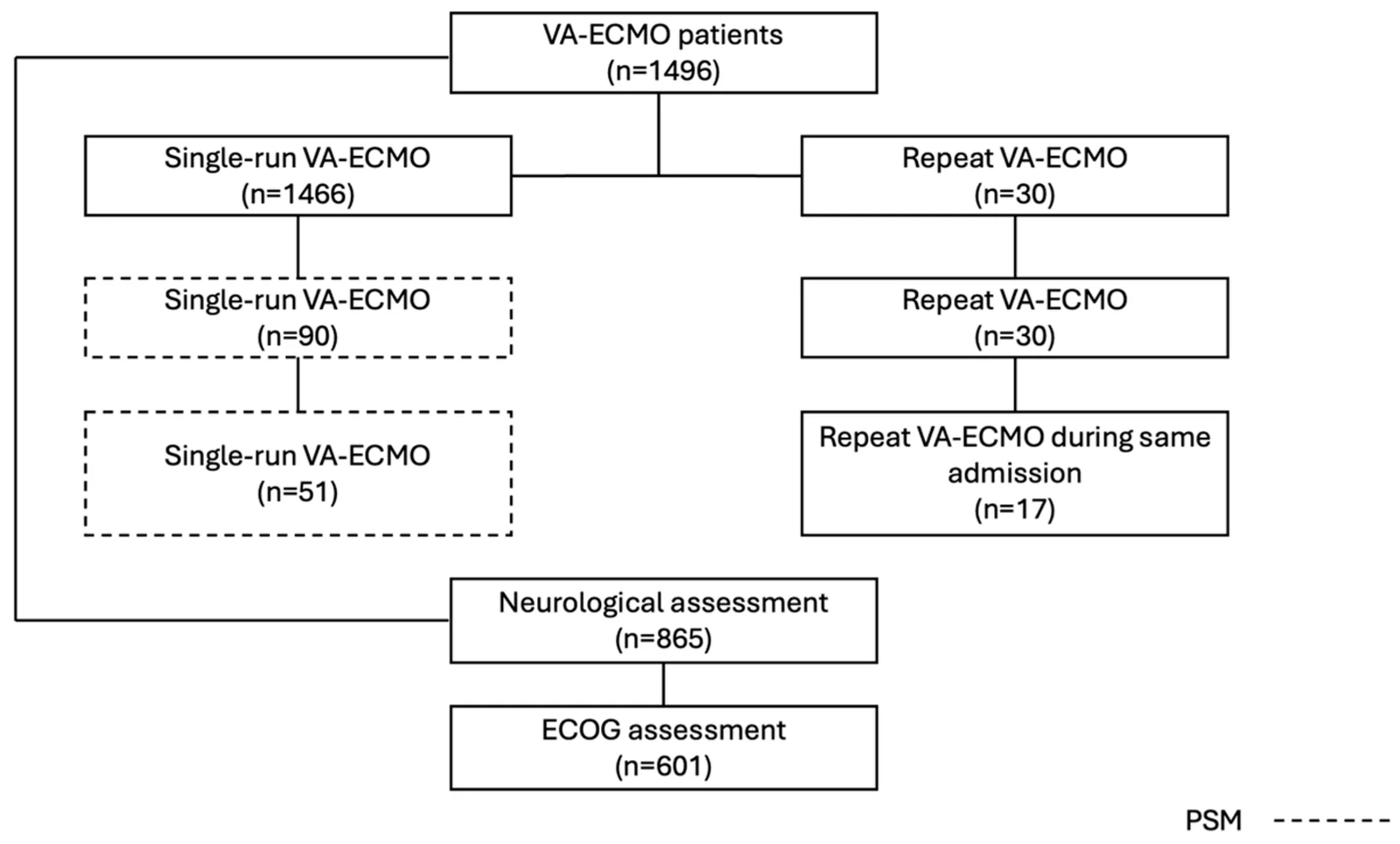
Flowchart explaining the study design.

This observational, single-center, retrospective study was conducted to evaluate adult patients (≥18 years) who underwent a second run of veno-arterial extracorporeal membrane oxygenation (VA-ECMO) between 2006 and 2024 (Figure 1).

Clinical outcomes were compared between patients who required only one VA-ECMO run and those who underwent a second VA-ECMO run. The primary endpoints included in-hospital mortality, 30-day outcomes, overall survival, and functional status of patients. Functional status was evaluated using the Eastern Cooperative Oncology Group (ECOG) performance status, which was assessed exclusively in hospital survivors. The initial ECOG assessment was performed at hospital discharge. Follow-up ECOG evaluations were routinely collected by the ECMO center coordinator from outpatient records, available post-discharge clinical documentation, and, when required, through structured telephone follow-up. All assessments were documented in the institutional database together with the date of evaluation. Due to the retrospective nature of this study, follow-up intervals and duration were not standardized and varied between patients.

Secondary exploratory analyses focused on differences between the two cohorts in baseline hemodynamic parameters, metabolic status, the development of multiorgan failure, neurological complications, and cannulation-related access-site complications, including overall access-related complications, cannulation-related bleeding events, limb ischemia attributable to cannulation, and access-site complications requiring subsequent surgical or interventional management.

ECMO decannulation decisions were based on hemodynamic stability, echocardiographic assessment of cardiac function, inotropic requirements to maintain cardiac output, arterial blood gas analysis, and markers of end-organ perfusion. Decannulation was performed following a joint decision by an intensivist and a cardiac surgeon once recovery sufficient to obviate the need for mechanical support was confirmed. During the study period, no uniform decannulation protocol was applied, as decannulation strategies varied over time and between cases. No weaning was attempted within 48 h after VA-ECMO initiation.

The decision to proceed with recannulation for a second VA-ECMO run was individualized and based on expert clinical judgment, following assessment of the likelihood of a favorable clinical prognosis. A second VA-ECMO run was defined as a new episode of VA-ECMO support initiated after successful weaning from VA-ECMO and decannulation at the end of the preceding ECMO course, followed by subsequent recannulation. Circuit exchanges or brief technical interruptions during otherwise continuous ECMO support were not classified as separate runs. Throughout the manuscript, the terms “second run,” “repeat VA-ECMO,” “ECMO re-initiation,” and “repeat support” refer to this same definition.

### 2.2. Definition of Organ Failure

All patients who had undergone VA-ECMO implantation suffered at least cardiocirculatory failure. In respiratory failure, diagnosis of ARDS was based on the Murray Score. A final score of more than 2.5 established the diagnosis of ARDS. Acute renal failure was defined by the parameters of the Kidney Disease Improving Global Outcomes (KDIGO) Clinical Practice Guidelines for AKI, namely Stage 3 of KDIGO criteria. The simultaneous occurrence of an increase in spontaneous INR to more than 1.5, a threefold increase in transaminases, and an increase in bilirubin to more than 2.5 mg/dL indicated acute hepatic dysfunction. Multiorgan failure was defined as the simultaneous dysfunction of three or more organ systems. Further details regarding the definitions and assessment of organ failure are provided in our previous study [13].

The neurological status was assessed in a subgroup of 881 patients eligible for neurological assessment.

### 2.3. Statistical Methods

The collected data were analyzed using IBM SPSS 31 (SPSS, Inc., Chicago, IL, USA) and SigmaPlot 15.0 (Systat Software Gmbh, Erkrath, Germany). A *p*-value ≤ 0.05 was considered statistically significant in all analyses.

To analyze differences between the two groups, the Mann–Whitney U test was used for nonparametric continuous variables, Student’s *t*-test for normally distributed continuous variables, and the chi-square test for categorical variables.

To evaluate a possible difference in survival, survival analysis using the log-rank was provided.

To reduce baseline differences between groups, propensity score matching was performed in SPSS using nearest-neighbor matching with a 3:1 ratio, matching three patients with a single VA-ECMO run to one patient with a second VA-ECMO run, incorporating clinically relevant baseline covariates: age, body mass index, sex, serum lactate level prior to ECMO initiation, cardiac surgery before ECMO initiation, and cardiopulmonary resuscitation prior to ECMO. A caliper width of 0.2 standard deviations of the logit of the propensity score was applied.

## 3. Results

A total of 1496 adult patients who underwent VA-ECMO were analyzed from a prospectively maintained database at a tertiary-care center between 2006 and 2024. Among these, 30 patients (2.0%) required repeat extracorporeal life support and constituted the second-run cohort.

The indications for re-initiation of VA-ECMO were heterogeneous. Low cardiac output (LCO), including both cardiogenic shock and postcardiotomy cardiogenic shock, was the most frequent indication, accounting for 17 patients (56.7%). Primary graft failure (PGF) following heart transplantation (HTx) was observed in six patients (20.0%). Refractory arrhythmia necessitated repeat VA-ECMO support in three patients (10.0%), while shock due to pulmonary embolism was identified in three patients (10.0%). Septic shock was the indication for repeat VA-ECMO in one patient (3.3%).

Multiorgan failure was the leading cause of in-hospital mortality in the second-run cohort (seven patients, 38.9%), followed by refractory cardiogenic shock with persistent LCO (6 patients, 33.3%). Cerebral death occurred in four patients (22.2%), and one patient (5.6%) died from severe refractory respiratory failure due to irreversible lung injury.

Among the patients requiring a second VA-ECMO run, 17 patients (56.7%) underwent repeat ECMO during the same hospitalization, whereas 13 patients (43.3%) were discharged from the hospital before ECMO re-initiation.

The median time interval between termination of the first ECMO run and initiation of the second run was 31.5 days [interquartile range (IQR): 4.0–271.8] for the overall cohort. In patients who remained hospitalized, the interval was 4.0 days [3.5–12.5], compared with 286 days [112–592] in those discharged prior to repeat ECMO.

The overall median age was 60.1 years [IQR: 51.0–68.6], with no significant difference between first-run and second-run ECMO patients (60.3 vs. 57.2 years; *p* = 0.102). The proportion of female patients was comparable between groups (27.3% vs. 36.7%; *p* = 0.254). Similarly, body mass index (BMI) did not differ significantly, with an overall median of 27.0 kg/m^2^ (27.0 vs. 25.7 kg/m^2^; *p* = 0.14; Table 1).

Peripheral cannulation via the femoral vein and artery was used in 91.3% of patients, with no significant difference between first-run and second-run ECMO cohorts (91.3% vs. 90.0%; *p* = 0.797). In contrast, cardiopulmonary resuscitation (CPR) prior to ECMO initiation was significantly more frequent in first-run patients (68.0% vs. 46.7%; *p* = 0.013), whereas postcardiotomy shock was more prevalent in the second-run cohort (27.1% vs. 53.3%; *p* = 0.002).

Patients requiring a repeat VA-ECMO procedure had a longer duration of mechanical circulatory support during the second run than those undergoing a single ECMO run (median 6 vs. 4 days; *p* = 0.003). In contrast, ECMO duration did not differ significantly between survivors and patients with in-hospital mortality (median 5 [3–12] vs. 7 [3.5–10.5] days; *p* = 0.915).

**Figure 2:**
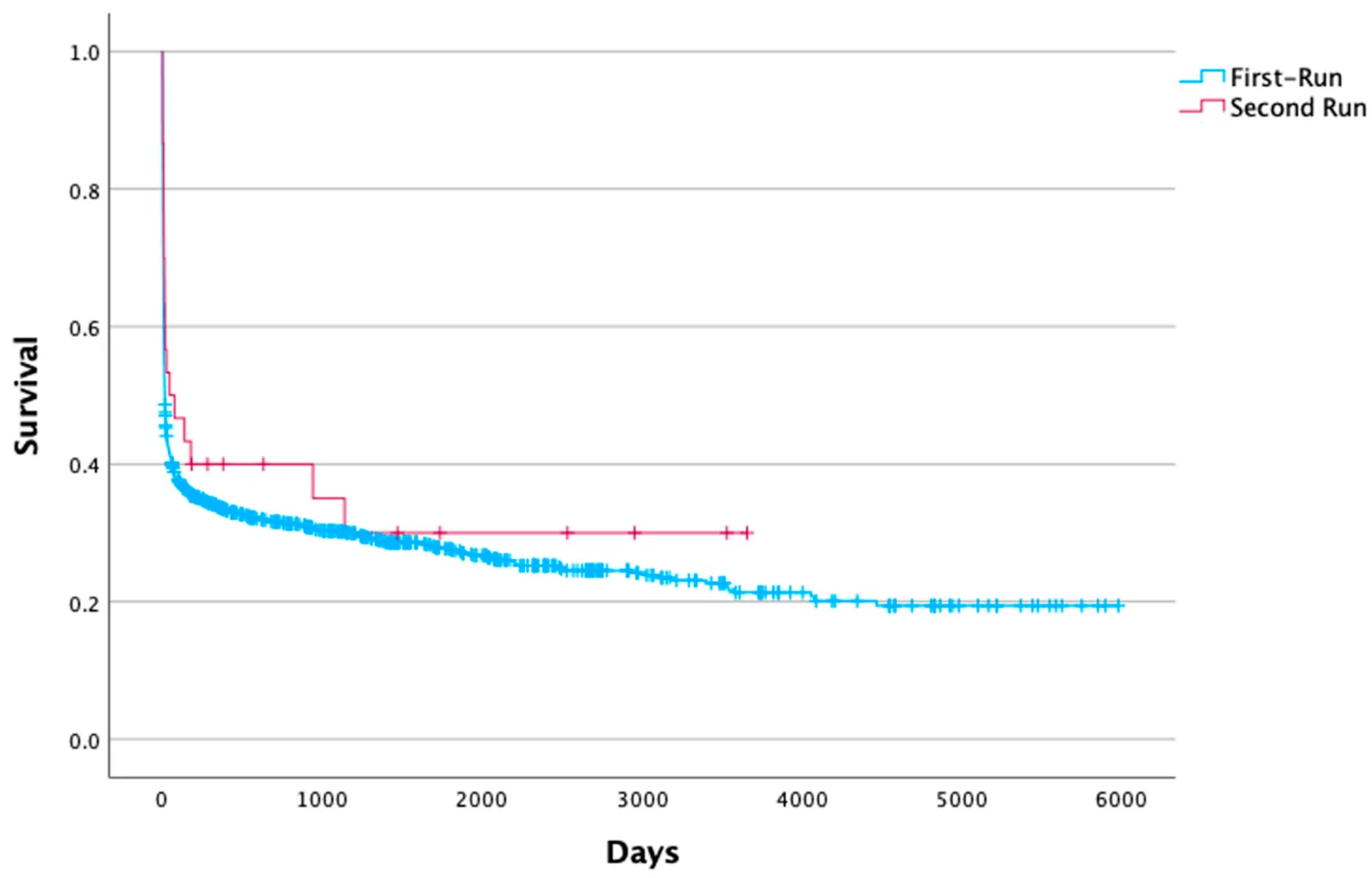
Kaplan–Meier estimation of survival over follow-up time between first-run and second-run groups.

An initial survival analysis during a median follow-up of 14 days (14.0 vs. 59.5 days; *p* = 0.097) revealed no significant differences between the two groups. In-hospital mortality was also not significantly different between groups (OR 1.01; 95% CI, 0.48–2.11; *p* = 0.984). Similarly, 30-day survival (OR 1.49; 95% CI, 0.72–3.07; *p* = 0.280) and overall survival (OR 1.27; 95% CI, 0.59–2.73; *p* = 0.272) did not differ significantly between groups (Figure 2). In addition, ECMO weaning success did not differ significantly between first-run and second-run patients (OR 1.76; 95% CI, 0.80–3.88; *p* = 0.153).

Outcomes according to the indication for repeat VA-ECMO are summarized descriptively in Table A1. Survival varied across indications, although the small subgroup sizes precluded meaningful inferential comparisons.

Functional status, assessed using the ECOG performance scale, was evaluated in 601 hospital survivors and prospectively updated within the institutional database over 18 years, with a median follow-up of 952 [275–2072] days, but no evidence of a significant difference in ECOG performance status was observed between the cohorts (*p* = 0.098).

However, multiorgan failure (MOF) was significantly more prevalent in patients undergoing a second ECMO run than among first-run patients (23.3% vs. 8.1%; *p* = 0.003). None of the patients who subsequently underwent a second ECMO run had developed MOF during the first ECMO run. In contrast, among the seven patients undergoing a second ECMO run who developed multiorgan failure during ECMO, in-hospital mortality was 100%.

Neurological outcomes were assessed in a subgroup of 881 patients (865 first-run and 16 second-run patients) eligible for neurological evaluation. A favorable neurological outcome (CPC 1–2) was observed in 66.4% of the overall cohort (66.2% vs. 75.0% in the first-run and second-run groups, respectively; *p* = 0.644). Severe neurological impairment (CPC 3–4) was observed in 1.85% of first-run patients and in none of the second-run patients. Cerebral death (CPC 5) occurred in 31.9% and 25.0% of first-run and second-run patients, respectively (*p* = 0.703).

The baseline hemodynamic and metabolic profiles were largely comparable between the cohorts. However, a significant difference in early metabolic stabilization was observed 24 h after ECMO initiation, with higher lactate levels on day 1 in first-run patients than in the second-run cohort (34 vs. 19 mg/dL; *p* = 0.003).

Furthermore, patients requiring a second ECMO run demonstrated significantly higher transfusion requirements during support than those supported only once. Median exposure to red blood cell concentrates was 2 vs. 7.5 units (*p* < 0.001), 0 vs. 1 unit for platelet concentrates (*p* = 0.001), and 0 vs. 2 units for fresh frozen plasma (*p* = 0.006). In contrast, baseline hemoglobin concentrations were significantly higher in first-run patients (10.2 vs. 9.15 g/dL; *p* < 0.001).

**Figure 3:**
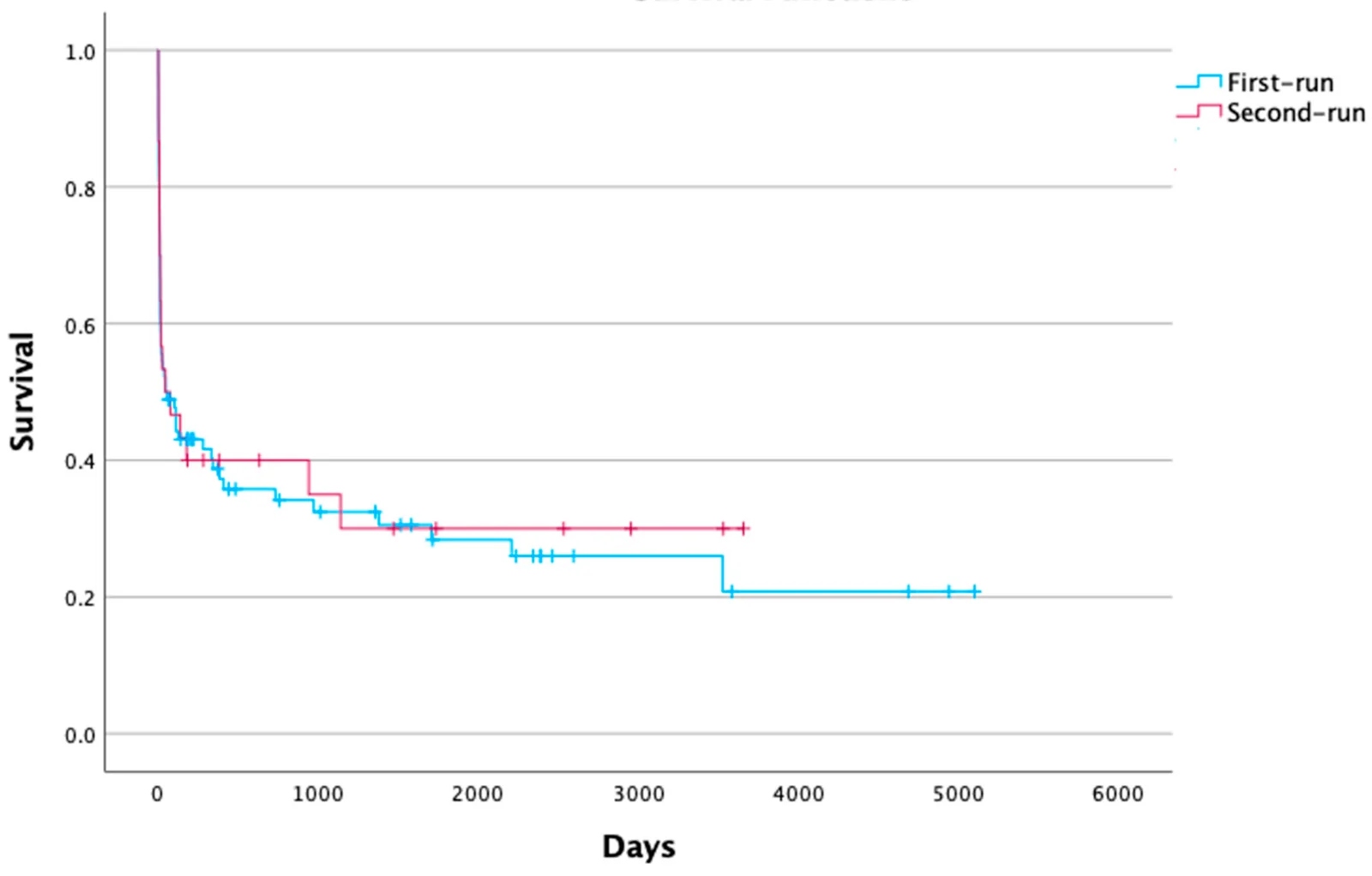
Kaplan–Meier estimation of survival over follow-up time between first-run and second-run groups after propensity score matching.

After propensity score matching to reduce baseline heterogeneity, 120 patients (90 single-run vs. 30 repeat VA-ECMO patients) were included in the analysis. Patients undergoing repeat ECMO demonstrated a longer duration of circulatory support during the second run than first-run patients, with a median of 6 vs. 4 days (*p* = 0.038). The incidence of multiorgan failure remained significantly higher in the second-run cohort (23.3% vs. 5.6%; *p* = 0.005). In addition, patients requiring repeat circulatory support continued to show greater transfusion requirements, including red blood cell concentrates (median 7.5 vs. 3 units; *p* = 0.011), platelet concentrates (1 vs. 0 unit; *p* = 0.028), and fresh frozen plasma (2 vs. 0 units; *p* = 0.050; Table 2). Despite these differences, in-hospital mortality, 30-day survival, and overall survival did not differ significantly between the matched cohorts (Figure 3). In addition, no significant difference in ECOG functional status was observed (*p* = 0.882).

**Figure 4:**
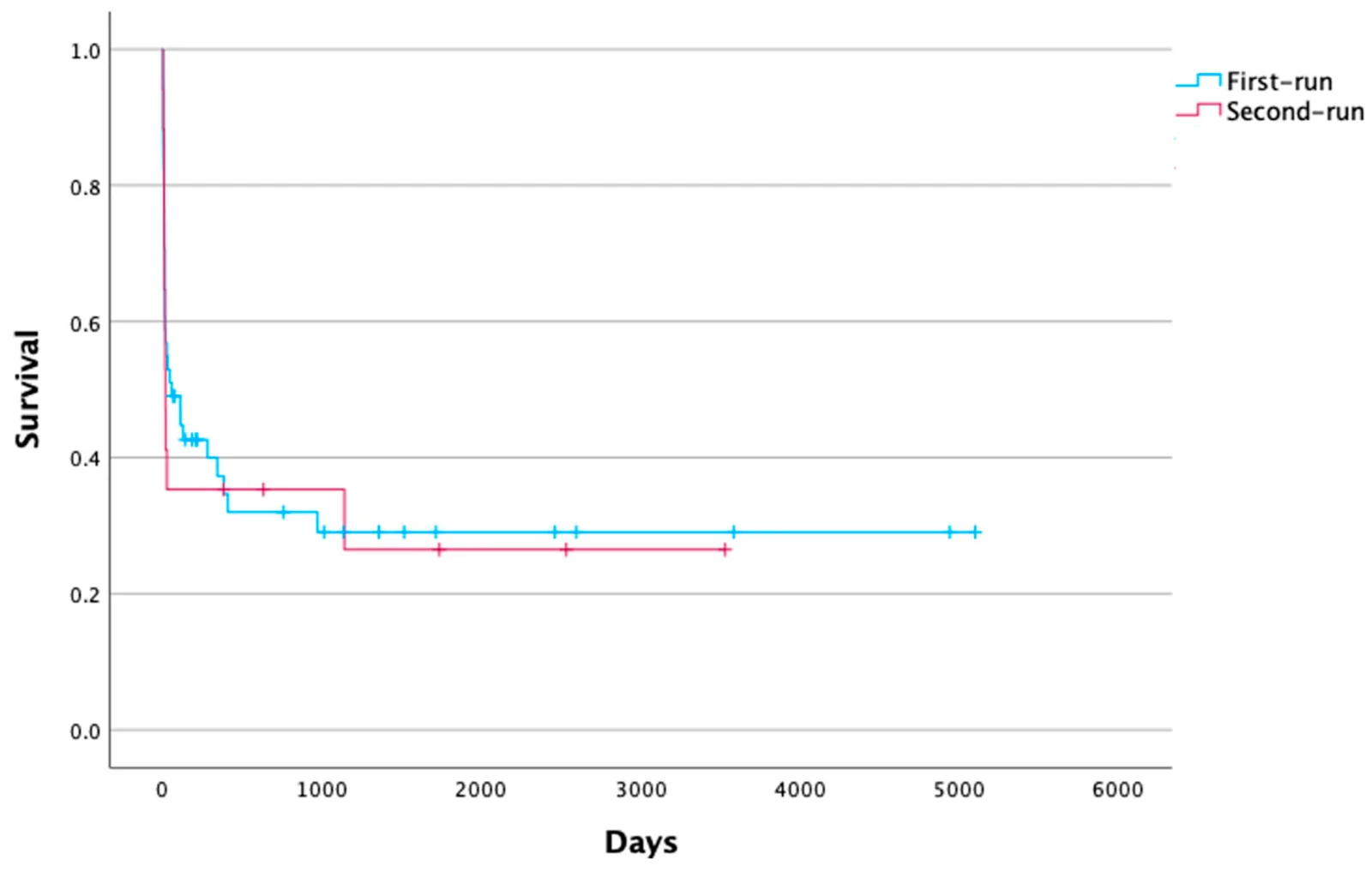
Kaplan–Meier estimation of survival over follow-up time between first-run and second-run groups of patients undergoing VA-ECMO within the same in-hospital stay after propensity score matching.

To further explore outcomes in patients requiring repeat circulatory support during the same admission, a subgroup analysis was performed. Given the smaller size of the repeat VA-ECMO subgroup, propensity score matching was repeated to ensure adequate comparability between groups, which included 68 patients overall (51 single-run and 17 repeat-run patients). Even within this selected population, patients undergoing a second ECMO run consistently required prolonged mechanical support (median 7 vs. 4 days; *p* = 0.039) and exhibited a higher incidence of multiorgan failure (23.5% vs. 5.9%; *p* = 0.038). Notably, these differences were not accompanied by excess mortality, as in-hospital, 30-day, and overall survival did not differ significantly between groups (Table 3; Figure 4). However, second-run survivors demonstrated numerically worse functional status (median ECOG 2 vs. 1; *p* = 0.057). Given the limited sample size and an approximate achieved power of 41.3%, the absence of conventional statistical significance should not be interpreted as evidence of equivalent functional recovery, and a clinically relevant difference cannot be excluded. Nevertheless, patients requiring repeat ECMO support continued to demonstrate a greater need for transfusion support, particularly red blood cell concentrates (median 8 vs. 3 units; *p* = 0.05).

In addition, ECMO cannulation-related access-site complications were evaluated, including overall access-related complications, cannulation-related bleeding events, limb ischemia attributable to cannulation, and access-site complications requiring subsequent surgical or interventional management. Both before and after propensity score matching, including the analysis of patients undergoing ECMO within the same in-hospital stay, no statistically significant differences were observed between the groups across all access-site-related endpoints (Table 4).

## 4. Discussion

With the expanding clinical use of ECLS, an increasing number of patients are being considered for VA-ECMO re-initiation [11,14]. Nevertheless, evidence regarding repeat ECMO support remains limited and heterogeneous, and reported outcomes are variable. Most published series comprise small case numbers and highly heterogeneous patient populations [9,12]. In several studies, survival analyses pooled veno-venous and veno-arterial ECMO cohorts, generally demonstrating inferior outcomes following repeat ECMO compared with those in patient cohorts with only one run [11,14]. In contrast, studies restricted to VA-ECMO populations, particularly those including patients with postcardiotomy cardiogenic shock, have reported outcomes comparable to those observed in the present study [8,9,10]. In our single-center VA-ECMO cohort, repeat ECMO support was associated with higher morbidity, but not with increased short- or long-term mortality, even after adjustment and in a same-admission subgroup.

With regard to functional outcomes, no significant differences were observed between groups in the unadjusted analysis, despite substantial baseline heterogeneity, particularly with respect to postcardiotomy and eCPR status. This finding persisted after adjustment using PSM. However, in a subgroup analysis restricted to patients undergoing repeat ECMO during the same index hospitalization, a trend toward impaired ECOG performance was observed among second-run patients, although it did not reach statistical significance, possibly due to the limited statistical power of the subgroup analysis. Furthermore, the longer duration of ECMO support observed in second-run patients, despite the absence of statistically significant differences in survival, may reflect a greater proinflammatory burden and increased vulnerability to multiorgan dysfunction during prolonged extracorporeal support, as suggested by previous evidence linking inflammatory activation to adverse outcomes during ECMO [13]. The increased incidence of multiorgan failure may also reflect the cumulative burden of recurrent circulatory failure, coagulopathy, and repeated transfusion exposure. Although causality cannot be established from these observational data, the findings suggest that systemic deterioration, rather than mortality alone, may represent an important limitation of repeat VA-ECMO and should be considered in patient selection and ongoing reassessment. Multiorgan failure emerged as a major limiting factor, as none of the patients who developed MOF during the second ECMO run survived to hospital discharge. Thus, the development of MOF during repeat support may identify a particularly high-risk stage of the clinical course.

Interestingly, lactate concentrations at 24 h were lower in patients undergoing repeat VA-ECMO despite comparable vasoactive support. This observation may reflect differences in patient selection, timing of ECMO re-initiation, or more rapid restoration of systemic perfusion. Given that lactate kinetics are influenced by both production and clearance, this finding should be interpreted accordingly.

Another notable finding was the increased requirement for blood transfusions among patients undergoing a second ECMO run. This trend persisted after PSM and was observed both in patients who were discharged prior to ECMO re-initiation and in those who required repeat support during the same index hospitalization. Importantly, this increased transfusion demand was not accompanied by higher levels of markers of hemolysis or a higher incidence of cannulation-related bleeding complications. Similar observations of increased blood product utilization in repeat ECMO cohorts have been reported in previous studies [10]. This finding may be attributable to the cumulative effects of prolonged critical illness and repeated extracorporeal support, including sustained systemic inflammatory activation, endothelial dysfunction [15], and ECMO-associated coagulopathy [16], which may increase transfusion requirements independent of overt bleeding or hemolysis. In addition, repeated exposure to anticoagulation, subclinical microvascular bleeding, and inflammation-driven alterations in hemostatic balance may contribute to higher transfusion needs in this population [15,17]. This observation may be clinically relevant, as transfusion burden itself has been associated with adverse outcomes and may further amplify inflammatory and organ dysfunction pathways [18].

No significant differences were observed in cannulation-related complications, including bleeding, ischemic events, or access-site complications requiring intervention. This may reflect careful patient selection and a structured cannulation strategy in patients undergoing repeat ECMO in our tertiary-care center.

The findings of the present study contribute important evidence to the ongoing clinical challenge of balancing the potential benefits of a second VA-ECMO run against the risks associated with prolonged mechanical circulatory support and futile outcomes. While overall survival did not differ significantly between first-run and second-run patients, our data provide the first systematic insight into post-support functional outcomes in this population, demonstrating a trend toward poorer functional recovery among second-run patients that did not reach statistical significance (*p* = 0.058). Importantly, repeat ECMO was associated with an increased risk of multiorgan failure, which appears to represent a major limiting factor for favorable outcomes.

Although propensity score matching reduced important baseline differences, residual confounding by indication cannot be excluded, particularly given the heterogeneity of VA-ECMO indications and the limited size of the repeat-ECMO cohort. These possible limitations underscore the need for larger, multicenter cohorts to validate our findings, achieve sufficient statistical power, and enable more robust analyses, including meta-analyses. Such evidence is essential to inform future guidelines and to support clinicians in the complex decision-making process surrounding repeat ECMO initiation [10]. At present, these decisions remain largely individualized and based on expert judgment, anticipated reversibility, and expected prognosis, which introduces inherent selection bias and limits reliable estimation of treatment-related risks and outcomes. Systematic risk stratification and earlier, structured reassessment of clinical trajectories may therefore help identify patients most likely to benefit from continued or alternative mechanical circulatory support.

At the same time, in patients with persistently poor prognostic indicators and limited prospects for meaningful recovery, timely interdisciplinary discussions regarding goals of care and proportionality of treatment should be encouraged. In selected cases, this may include consideration of treatment de-escalation to prevent prolonged non-beneficial interventions and excessive utilization of healthcare resources.

### Limitations

The long study period of 18 years encompasses substantial changes in ECMO technology and clinical management, which may have influenced outcomes; however, the small number of repeat VA-ECMO patients precluded reliable era-stratified analyses. This study’s retrospective observational design limits causal inference and is prone to selection bias. In addition, the absence of uniform protocols for ECMO weaning and repeat-run inclusion and initiation may have introduced variability in clinical decision-making. The relatively small number of patients undergoing repeat ECMO, with an inherent risk of type II error (beta error), together with the heterogeneity of the study population, may limit the conclusiveness of our findings. In addition, patients undergoing repeat VA-ECMO necessarily survived the first ECMO course and remained candidates for further intensive treatment, introducing inherent survivor selection bias that cannot be fully addressed by propensity score matching. The ECOG analysis was limited to hospital survivors with available follow-up data, introducing potential survivor and availability bias.

## 5. Conclusions

Repeat VA-ECMO may represent a feasible therapeutic option in carefully selected patients, without statistically significant differences in survival compared with single-run VA-ECMO. However, repeated support is accompanied by prolonged VA-ECMO duration and an increased incidence of multiorgan failure. In patients undergoing repeat VA-ECMO during the same hospital stay, potentially unfavorable functional outcomes cannot be excluded. Larger, prospective, multicenter studies are required to further clarify the clinical implications of repeat ECMO.

## Figures and Tables

**Table 1:** Baseline characteristics of the study population.

Category	All Patients	First Run	Second Run	*p*-Value
Number of patients	1496	1466	30	
Age, years	60.1 [51.0; 68.6]	60.25 [51.27; 68.825]	57.2 [48.75; 63.125]	0.102
Women n, (%)	411 (27.5%)	400 (27.3%)	11 (36.7%)	0.254
BMI, kg/cm^2^	27.0 [24.2; 30.1]	27.0 [24.2; 30.1]	25.65 [22.2; 29.6]	0.140
Peripheral ECMO canulation	1366 (91.3%)	1339 (91.3%)	27 (90%)	0.797
ECMO weaned	856 (57.2%)	835 (57.0%)	21 (70%)	0.153
Follow-up, days	14 [3; 537.5]	14 [3; 531.5]	59.5 [7.75; 989.75]	0.097
CPR before ECMO, patients	1011 (67.6%)	997 (68.0%)	14 (46.7%)	**0.013**
Postcardiotomy patients	414 (27.7%)	398 (27.1%)	16 (53.3%)	**0.002**
ECOG; n, number of patients	1 [1; 1]; n = 601	1 [1; 1]; n = 589	1 [1; 2]; n = 12	0.098
Days of ECMO	4 [2; 7]	4 [2; 7]	6 [3; 11.25]	**0.003**
In-hospital mortality	895 (59.8%)	877 (59.8%)	18 (60%)	0.984
30 days survival	653 (43.6%)	637 (43.5%)	16 (53.3%)	0.28
Overall survival	425 (28.4%)	415 (28.3%)	10 (33.3%)	0.272
Distal perfusion	511 (34.2%)	498 (33.0%)	13 (43.3%)	0.284
CVVHD	602 (40.2%)	586 (40.0%)	16 (53.3%)	0.14
Number of failure organs	1 [1; 2]	1 [1; 2]	2 [1; 2.25]	**0.026**
Multiorgan failure	126 (8.4%)	119 (8.1%)	7 (23.3%)	**0.003**
***Neurological outcome between 881 patients (865 patients from first-run group und 16 patients from second-run group)***				
CPC 1–2	585 (66.4%)	573 (66.2%)	12 (75%)	0.644
CPC 3–4	16 (1.8%)	16 (1.85%)	0 (0%)	0.717
CPC 5	280 (31.8%)	276 (31.9%)	4 (25%)	0.703
***Baseline hemodynamic and metabolic parameters***				
VIS before ECMO	34.13 [12.82. 64.89]	34.484 [12.72; 65.2]	31.373 [17.81; 52.05]	0.680
VIS 1st day	16.67 [0; 46.67]	16.04 [0; 46.67]	33.492 [0.0; 47.76]	0.315
Lactate before ECMO. mg/dL	81 [45; 123]	81.5 [46; 123.75]	56.5 [28.75; 115]	0.051
Lactate 1st day. mg/dL	33 [17; 68]	34 [17.5; 68.5]	19 [11; 41]	**0.003**
MAP before ECMO, mmHg	51 [40; 62]	51 [40; 62]	57 [47.25; 65.25]	0.157
MAP 1st day, mmHg	65 [60: 71]	65 [60; 71]	68 [61; 72]	0.603
arterial pH before ECMO	7.23 [7.12; 7.32]	7.23 [7.12; 7.32]	7.22 [7.13; 7.36]	0.542
arterial pH 1st day in ECMO	7.41 [7.36; 7.47]	7.41 [7.36; 7.47]	7.42 [7.39; 7.48]	0.274
***Laboratory parameters and blood product utilization***				
Hb before ECMO, g/dL	10.1 [8.6; 12]	10.2 [8.6; 12]	9.15 [7.2; 10.2]	**<0.001**
Hb 1st day, g/dL	9.5 [8.9; 10.7]	9.6 [8.9; 10.7]	9 [8.4; 9.5]	**0.003**
Thrombocytes before ECMO/nl	171.5 [119; 236.75]	173.5 [121; 236.25]	123 [90.25; 258.5]	0.108
Thrombocytes 1st day/nl	119 [80; 163]	119 [80; 163]	118 [85; 168]	0.736
Transfusion of ECs during ECMO, units	2 [0; 7]	2 [0; 7]	7.5 [2; 15]	**<0.001**
Transfusion of TCs during ECMO, units	0 [0; 1]	0 [0: 1]	1 [0; 6]	**0.001**
Transfusion of FFPs during ECMO, units	0 [0; 4]	0 [0; 4]	2 [0; 10.25]	**0.006**
free Hb before ECMO, mg/L	181 [67; 422.5]	193 [70; 432]	97.5 [51; 147.25]	**0.004**
Highest level of free Hb during ECMO, mg/L	274 [124; 560.5]	275 [125; 560]	196 [103; 594.5]	0.493
Highest level of NSE during ECMO, μg/L	58 [38.25; 101]	58 [38; 102]	55 [38.5; 92]	0.607

Continuous variables are presented as median [interquartile range] and categorical variables as number (percentage).

**Table 2:** Baseline characteristics of the study population after propensity score matching.

Category	All Patients	First Run	Second Run	*p*-Value
Number of patients	120	90	30	
Age, years	57.5 [47.4; 65.65]	56.0 [43.05; 67.175]	56.5 [47.7; 63.0]	0.966
Women, n (%)	35 (29.2%)	24 (26.7%)	11 (36.7%)	0.297
BMI kg/cm^2^	25.47 [23.44; 28.6]	25.47 [23.8; 28.25]	25.69 [22.23; 29.6]	0.785
ECMO weaned	80 (66.7%)	59 (65.6%)	21 (70%)	0.655
Follow-up, days	50.5 [7; 706]	50.5 [6.75; 544.25]	59.5 [7.75; 989.75]	0.66
CPR before ECMO	49 (40.8%)	35 (38.9%)	14 (46.7%)	0.453
Days of ECMO	4.5 [2; 8]	4 [2; 7]	6 [3; 11.25]	**0.038**
ECOG; n, number of patients	1 [1; 2], n = 57	1 [1; 2]; n = 45	1 [1; 2]; n = 12	0.882
In-hospital mortality	63 (52.5%)	45 (50%)	18 (60%)	0.342
30 days survival	56 (46.7%)	42 (46.7%)	14 (46.7%)	1.0
Overall survival	38 (31.7%)	28 (31.1%)	10 (33.3%)	0.821
CVVHD	56 (46.6%)	40 (44.4%)	16 (53.3%)	0.398
Multiorgan failure	12 (10%)	5 (5.6%)	7 (23.3%)	**0.005**
***Neurological outcome between 76 patients (60 patients from first-run group und 16 patients from second-run group)***				
CPC 1–2	56 (73.7%)	44 (73.3%)	12 (75%)	0.419
CPC 3–4	2 (2.6%)	2 (3.3%)	0 (0%)	0.332
CPC 5	18 (23.7%)	14 (23.3%)	4 (25%)	0.419
***Baseline hemodynamic and metabolic parameters***				
VIS before ECMO	35.49 [16.43; 64.1]	36.44 [15.26; 78.62]	31.37 [17.81; 52.05]	0.301
VIS 1st day	25.13 [2.48. 50]	21.67 [2.68; 53.04]	33.49 [0; 47.76]	0.793
Lactate before ECMO, mg/dL	59.5 [31; 96.75]	62.5 [33; 95]	56.5 [28.75; 115.0]	0.935
Lactate 1st day, mg/dL	25 [15; 58.5]	29 [16; 62]	19 [11; 41]	0.025
MAP before ECMO, mmHg	56 [45; 67]	56 [41; 67.75]	58 [50; 65]	0.940
MAP 1st day, mmHg	66 [60.5; 72]	65 [60; 72]	68 [61; 72]	0.889
Arterial pH before ECMO	7.25 [7.18; 7.36]	7.26 [7.18; 7.36]	7.22 [7.13; 7.36]	0.622
Arterial pH 1st day in ECMO	7.42 [7.39; 7.48]	7.415 [7.38; 7.49]	7.42 [7.39; 7.48]	0.737
***Laboratory parameters and blood product utilization***				
Hb before ECMO	9.5 [8.025; 10.4]	9.5 [8.475; 11]	9.15 [7.2; 10.2]	**0.017**
Hb 1st day	9.4 [8.75; 10.5]	9.65 [8.875; 10.8]	9 [8.4; 9.5]	**0.002**
Thrombocytes before ECMO	162 [108; 251]	167 [114.5; 247]	123 [90.25; 258.5]	0.168
Thrombocytes 1st day	118 [87; 163]	118.5 [87; 159.75]	118.5 [87; 159.75]	0.972
Transfusion of ECs during ECMO, units	4.5 [0; 9.75]	3 [0; 9]	7.5 [2; 15]	**0.011**
Transfusion of TCs during ECMO, units	0 [0; 2]	0 [0; 2]	1 [0; 6]	**0.028**
Transfusion of FFPs during ECMO, units	0 [0; 6]	0 [0; 4]	2 [0; 10.25]	**0.05**
Free Hb before ECMO, mg/L	113 [52; 345]	166 [47; 441.5]	97.5 [51; 147.25]	0.083
Highest level of free Hb during ECMO, mg/L	287 [130.5; 582.75]	294.5 [140.5; 575]	196 [103; 594.5]	0.392
Highest level of NSE during ECMO, μg/L	54 [33; 84.5]	52 [32; 77]	55 [38.5; 92]	0.473

Continuous variables are presented as median [interquartile range] and categorical variables as number (percentage).

**Table 3:** Characteristics of the study population undergoing VA-ECMO within the same in-hospital stay after propensity score matching.

Category	All Patients	First Run	Second Run	*p*-Value
Number of patients	68	51	17	
Age, years	57.0 [46.78; 66.25]	55.9 [39.3; 67.3]	58.3 [50.0; 63.5]	0.374
Women n. (%)	22 (32.4%)	14 (27.5%)	8 (47.1%)	0.134
BMI kg/cm^2^	26.2 [23.4; 29.075]	26.1 [23.4; 27.8]	26.2 [23.4; 33.25]	0.263
ECMO weaned	42 (61.8%)	32 (62.7%)	10 (58.8%)	0.773
Follow-up, days	26.5 [7; 575.25]	56 [6; 408]	15 [7.5; 885]	0.960
CPR before ECMO	30 (44.1%)	22 (43.1%)	8 (47.1%)	0.778
ECOG; n, number of patients	1 [1; 2]; n = 23	1 [1; 1.5]; n = 17	2 [1; 2]; n = 6	0.057
Days of ECMO	5.5 [2; 9.5]	4 [2; 7]	7 [4; 12]	**0.039**
30 days survival,	33 (48.5%)	27 (52.9%)	6 (35.3%)	0.207
First year survival, day	26 (38.2%)	20 (39.2%)	6 (35.3%)	0.773
Overall survival, days	22 (32.5%)	17 (33.3%)	5 (29.4%)	0.765
Distal perfusion	22 (32.5%)	15 (29.4%)	7 (41.2%)	0.369
CVVHD	31 (45.6%)	21 (41.2%)	10 (58.8%)	0.206
Multiorgan failure	7 (10.3%)	3 (5.9%)	4 (23.5%)	**0.038**
***Neurological outcome between 42 patients (34 patients from first-run group and 8 patients from second-run group)***				
CPC 1–2	31 (73.8%)	25 (71.4%)	6 (75%)	0.353
CPC 3–4	1 (2.38%)	1 (2.9%)	0 (0%)	0.321
CPC 5	10 (23.8%)	8 (23.5%)	2 (25%)	0.353
***Baseline hemodynamic and metabolic parameters***				
VIS before ECMO	35.486 [13.886; 60.588]	35.556 [12.626; 64.103]	34.848 [21.458; 58.17]	0.960
VIS 1st day	22.902 [2.48; 47.99]	20 [2.381; 50]	34.762 [3.745; 45.98]	0.517
Lactate before ECMO, mg/dL	60.5 [33; 101.5]	70 [34; 103]	39 [27; 79.5]	0.068
Lactate 1st day, mg/dL	25.5 [12.75; 51]	29 [16; 58.5]	15 [9; 25]	**0.013**
MAP before ECMO, mmHg	59 [39.5; 68]	56 [38; 68]	61 [51; 66]	0.323
MAP 1st day, mmHg	65 [60.25; 71.75]	65 [60; 71.5]	69 [61; 72]	0.343
arterial pH before ECMO	7.235 [7.145; 7.36]	7.25 [7.16; 7.36]	7.22 [7.115; 7.405]	0.868
arterial pH 1st day in ECMO	7.435 [7.39; 7.49]	7.45 [7.39; 7.5]	7.41 [7.39; 7.48]	0.420
***Laboratory parameters and blood product utilization***				
Hb before ECMO	9.5 [8.35; 10.4]	9.5 [8.5; 10.5]	9.5 [7.6; 10.2]	0.318
Hb 1st day	9.5 [8.8; 10.4]	9.8 [8.9; 10.5]	9.3 [8.4; 9.6]	**0.025**
LDH before ECMO, U/L	486 [325.75; 804.25]	417.5 [290.75; 684]	661 [499.25; 1198.75]	**0.005**
LDH 1st day, U/L	540 [364; 1088]	519 [306.25; 911]	579 [465; 1746]	0.115
Transfusion of ECs during ECMO	4 [0; 10]	3 [0; 9]	8 [1.5; 16]	**0.05**
Transfusion of TCs during ECMO	0 [0; 2]	0 [0; 2]	0 [0; 5.5]	0.390
Transfusion of FFPs during ECMO	0 [0; 3.75]	0 [0; 6]	0 [0; 3]	0.909
Free Hb before ECMO, mg/L	156 [59.75; 378.25]	240.5 [78; 619.25]	97.5 [53.5; 144.75]	**0.006**
Highest level of free Hb during ECMO, mg/L	267 [143; 538]	294.5 [159.25; 575.75]	168 [107.5; 388.5]	0.245
Highest level of NSE during ECMO, μg/L	57.0 [32.5; 92.5]	57 [32; 85]	74 [38.5; 99.25]	0.222

Continuous variables are presented as median [interquartile range] and categorical variables as number (percentage).

**Table 4:** ECMO cannulation-related access-site complications.

Category	Patients	First Run	Second Run	*p*-Value
**Between all patients**				
overall access-related complications	361 (24.1%)	354 (24.2%)	7 (23.3%)	0.918
cannulation-related bleeding events	155 (10.4%)	151 (10.3%)	4 (13.3%)	0.587
limb ischemia attributable to cannulation	222 (14.8%)	219 (14.9%)	3 (10.0%)	0.457
access-site complications requiring subsequent surgical or interventional management	236 (15.8%)	232 (15.8%)	4 (13.3%)	0.711
**Between all patients after propensity score matching**				
overall access-related complications	32 (26.6%)	25 (27.8%)	7 (23.3%)	0.634
cannulation-related bleeding events	13 (10.8%)	9 (10.0%)	4 (13.3%)	0.611
limb ischemia attributable to cannulation	20 (16.7%)	17 (18.9%)	3 (10.0%)	0.258
access-site complications requiring subsequent surgical or interventional management	20 (16.7%)	16 (17.8%)	4 (13.3%)	0.572
**Patients undergoing VA-ECMO within the same in-hospital stay after propensity score matching**				
overall access-related complications	16 (23.5%)	12 (23.5%)	4 (29.4%)	1.0
cannulation-related bleeding events	7 (10.3%)	5 (9.8%)	2 (11.8%)	0.818
limb ischemia attributable to cannulation	10 (14.7%)	8 (15.7%)	2 (11.8%)	0.693
access-site complications requiring subsequent surgical or interventional management	10 (14.7%)	8 (15.7%)	2 (11.8%)	0.693

Categorical variables are presented as number (percentage).

## Data Availability

The datasets used and/or analyzed during the current study are available from the corresponding author on reasonable request.

## Abbreviations

The following abbreviations are used in this manuscript:

AKI	Acute kidney injury
ARDS	Acute respiratory distress syndrome
BMI	Body Mass Index
CPC	Cerebral Performance Category
CPR	Cardiopulmonary resuscitation
CVVHD	Continuous veno-venous hemodialysis
EC	Erythrocyte concentrate
ECLS	Extracorporeal life support
ECMO	Extracorporeal membrane oxygenation
ECOG	Eastern Cooperative Oncology Group
ELSO	Extracorporeal Life Support Organization
FFP	Fresh frozen plasma
Hb	Hemoglobin
HTx	Heart transplantation
INR	International normalized ratio
IQR	Interquartile range
KDIGO	Kidney disease: improving global outcomes
LCO	Low cardiac output
LDH	Lactate dehydrogenase
MAP	Mean arterial pressure
MOF	Multiorgan failure
NSE	Neuron-specific enolase
OR	Odds Ratio
PGF	Primary graft failure
PSM	Propensity score matching
RBC	Red blood cell
SPSS	Statistical Package for the Social Sciences
TC	Thrombocyte concentrate
VA-ECMO	Veno-arterial extracorporeal membrane oxygenation
VIS	Vasoactive-Inotropic Score
eCPR	Extracorporeal cardiopulmonary resuscitation

## Appendix A

**Table A1:** Outcomes according to the indication for repeat VA-ECMO.

Indication for Repeat VA-ECMO	n	ECMO Weaning, n (%)	In-Hospital Survival, n (%)	30-Day Survival, n (%)	Overall Survival, n (%)	MOF, n (%)
Low cardiac output/cardiogenic or postcardiotomy shock	17	10 (58.8)	7 (41.2)	8 (47.1)	5 (29.4)	5 (29.4)
Primary graft failure after HTx	6	6 (100)	3 (50.0)	6 (100)	3 (50.0)	2 (33.3)
Refractory arrhythmia	3	3 (100)	0 (0)	0 (0)	0 (0)	0 (0)
Pulmonary embolism	3	2 (66.7)	2 (66.7)	2 (66.7)	2 (66.7)	0 (0)
Septic shock	1	0 (0)	0 (0)	0 (0)	0 (0)	0 (0)
Overall	30	21 (70.0)	12 (40.0)	16 (53.3)	10 (33.3)	7 (23.3)

Data are presented as number (percentage). Given the small number of patients within individual indication subgroups, no inferential between-group comparisons were performed.
